# Acupuncture for thalamic pain after stroke: A systematic review and meta-analysis

**DOI:** 10.1097/MD.0000000000033006

**Published:** 2023-03-03

**Authors:** Wenfeng Li, Shaozong Chen

**Affiliations:** a School of Acupuncture and Moxibustion, Shandong University of Traditional Chinese Medicine, Shandong, China; b Acupuncture Research Institute of Shandong University of Traditional Chinese Medicine, Jinan, China.

**Keywords:** acupuncture, RCT, review, stroke, thalamic pain

## Abstract

To evaluate the efficacy and safety of acupuncture on thalamic pain after stroke.

**Methods:**

The self-established database was searched from 8 Chinese and English databases to June 2022, and the randomized controlled trials articles on the comparative treatment of thalamic pain after stroke with acupuncture were included. That visual analog scale, present pain intensity score, pain rating index, the total efficiency, and adverse reactions were mainly used to evaluate the outcomes.

**Results:**

A total of 11 papers were included. Meta-analysis showed that acupuncture appeared to be more effective than drugs for treatment of thalamic pain, as assessed by the visual analog scale [mean difference (MD) = −1.06, 95% confidence interval (CI) (−1.20, −0.91), *P* < .00001], the present pain intensity score [MD = −0.27, 95% CI (−0.43, −0.11), *P* = .001], the pain rating index [MD = −1.02, 95% CI (−1.41, −0.63), *P* < .00001], and the total efficiency [risk ratio = 1.31, 95% CI (1.22,1.41), *P* < .00001]. Meta-analysis results show that there is no significant difference in safety between acupuncture and drug therapy [risk ratio = 0.50, 95% CI (0.30,0.84), *P* = .009].

**Conclusion:**

Studies have shown that acupuncture in the treatment of thalamic pain is effective, and it does not prove to have a higher safety than drug treatment, therefore a large-scale multicenter randomized controlled trials study is needed to further prove.

## 1. Introduction

Stroke is a neurological disorder characterized by blockage of blood vessels and is the second leading cause of death globally, killing approximately around 5.5 million annually. The incidence of stroke in low-and-middle income countries is increasing year by year.^[1]^ Thalamic pain, also known as thalamic pain syndrome, which is a typical central poststroke pain (CPSP), often follows lateral medullary stroke and parietal cortical stroke and may develop anywhere along the spinothalamic or trigemino-thalamic pathways.^[2]^ Thalamic pain is characterized by thermal paresthesia, ectopic pain, and persistent pain.^[3]^ In previous reports on central pain cases, thalamic pain accounts for about 33% to 47%,^[4]^ mostly occurs within 3 to 6 months after stroke,^[5]^ and about 1% to 12% of stroke patients will experience thalamic pain within 1 year.^[6]^ Risk factors for development of CPSP include young age, previous depression, current smoking, and increased baseline stroke severity,^[7]^ Notably, studies have found that young stroke patients are twice as likely to develop CPSP.^[8]^ The disease severely reduces the quality of life, affects recovery, reduces sleep quality, and even leads to self-harm.^[3]^ At present, thalamic pain is mainly treated with drugs, including antidepressants, anticonvulsants, anticonvulsants, anesthetic agents, and analgesics.^[2]^ There are currently no meaningful preventive measures.^[3]^ Therefore, there is an urgent need to seek more efficient measures to treat or relieve this disease.

Acupuncture originated in China and is a treatment method of traditional Chinese medicine. It is guided by the theory of meridians and collaterals. Needles are inserted into specific parts or acupoints, and specific manipulations are applied to achieve the purpose of treating diseases. Traditional Chinese medicine attributes pain to the blockage of the meridians, “No general pain, general no pain,” Acupuncture acts on the whole rather than local areas, and for various acute and chronic pain, acupuncture has a good therapeutic effect.^[9–14]^

The thalamus is the relay station for all sensory information in the brain,^[15]^ the sensory information we receive in the environment goes from the peripheral nervous system to the central nervous system. when the information reaches the central nervous system, it reaches the thalamus, which has the function of decoding and processing information. The current findings suggest that patients are only likely to experience thalamic pain when thalamic lesions imply significant changes in the spinothalamic system.^[16]^ Therefore, the thalamus is thought to play an important role in the mechanism of central pain.^[17]^ Acupuncture analgesia achieves effect through nerve electrophysiological and biochemical changes under the premise of complete neural pathways.^[18]^ The essence is the integration process of the afferent impulses in the pain area and the afferent impulses in the acupoint area in the central nervous system.^[19]^ Acupuncture can inhibit the pain signals of the dorsal horn of the spinal cord and the medial thalamic nucleus, so that they cannot be transmitted, and achieve analgesic effect.^[20]^ Some neurons in the parafascicular nucleus and the central lateral nucleus of the thalamus can produce special unit discharges to some noxious stimuli, and this discharge can be canceled by morphine, and this discharge is related to pain perception. On the other hand, acupuncture analgesia may be mediated by the release of various endogenous neurotransmitters (enkephalin and dynorphin),^[14,21]^ regulation of the adrenergic system,^[22]^ accommodation of neurotransmitters such as 5-HT, deliver of various proinflammatory mediators.^[23]^

At present, there are few meta-analyses on acupuncture for thalamic pain.^[24,25]^ Therefore, this study evaluated the safety and efficacy of acupuncture versus medication in the treatment of thalamic pain based on scientific evidence.

## 2. Materials and methods

### 2.1. Search strategy

Eight Chinese and English databases including China National Knowledge Infrastructure Database, China Science and Technology Journal Database (VIP), Wanfang Database (CECDB), PubMed, Web of Science, Cochrane Library, Embase and karge were searched by computer, and the search time was from the establishment of the database to June 25, 2022. Using the combination of subject headings and free words, the retrieval strategy is adjusted according to the database. Taking PubMed as an example, the following key words were used for the literature search: (((((((((((((((Thalamic Diseases [MeSH Terms]) OR (thalamic pain)) OR (thalamic pain syndrome)) OR (central poststroke pain)) AND (stroke [MeSH Terms])) OR (Cerebrovascular Accident)) OR (CVA)) OR (Cerebrovascular Apoplexy)) OR (Brain Vascular Accident)) OR (Cerebrovascular Stroke)) AND (Acupuncture [MeSH Terms])) OR (Electroacupuncture [EA] [MeSH Terms]))) OR ((acupunct* [Title/Abstract])) OR (electro acupuncture)) AND ((Randomized Controlled Trial [Publication Type])) OR (random* [Title/Abstract])) OR (placebo [Title/Abstract]).

### 2.2. Article screening criteria

#### 1.2.2.. Inclusion criteria.

1. Randomized controlled (RCT) studies on the treatment of thalamic pain with acupuncture published at home or abroad.
2. The experimental groups were treated with acupuncture along with or without traditional Chinese medicine, and control groups were adopted drugs.
3. Acupuncture includes body acupuncture, scalp acupuncture, plum-blossom acupuncture, 3-edged acupuncture, electro-acupuncture, wrist-ankle acupuncture, etc.
4. Patients with thalamic pain at baseline were enrolled.
5. Outcome indicators: At least 1 clear indicator such as the total efficiency, visual analog scale (VAS), and adverse reactions is included.

#### 2.2.2. Exclusion criteria.

1. Repeated publications.
2. Non-acupuncture treatment in the treatment group, or nondrug treatment in the control group.
3. Articles with incorrect data or no relevant results.
4. Unable to retrieve full-text.
5. reviews, animal studies, case reports, dissertations.
6. cross-experimental design.

### 2.3. Outcomes

The primary outcomes were the total efficiency, the VAS, the present pain intensity score (PPI), the pain rating index (PRI), secondary outcomes included adverse reactions. Adverse events included systemic manifestations (asthenia), neurological manifestations (somnolence, dizziness, ataxia, dry mouth), gastrointestinal reactions (flatulence, nausea, gastrointestinal distress), cutaneous manifestations (urticaria, pruritus), liver and kidney damage and other adverse reactions.

### 2.4. Data extraction

Use Note Express 3.5.0 software to screen the retrieved articles and preliminarily exclude duplicate articles. Then the 2 researchers read the title and abstract of the article respectively. According to the inclusion and exclusion criteria, the articles that do not meet the requirements are excluded, and then the full text is read to confirm the inclusion. If there is a dispute, a third researcher will assist in the judgment and decide whether to include it or not after a joint discussion. Excel was used to extract the basic information of the incorporated articles, including; Basic information of the article (age, gender, course of disease, sample size, etc); Parameters of acupuncture (treatment frequency, course of treatment, needle retention time, etc); Intervention method, randomization plan, follow-up, adverse reactions, outcome indicators and other data.

### 2.5. Article quality evaluation

The Risk of Bias tool^[26]^ provided by Cochrane was used to assess the methodological quality of the included articles, including random sequence generation, allocation concealment, blinding of participants and personnel, blinding of outcome evaluation, incomplete outcome data, selective reporting, and other bias.

### 2.6. Statistical method

Meta-analysis was performed using the Cochrane systematic review software Review Manager 5.4. The involved articles were tested for heterogeneity. When *P* > .05 and *I*^2^ < 50%, the heterogeneity among the studies was considered to be small, and a fixed-effects model was used; on the contrary, when *P* ≤ .05 and *I*^2^ ≥ 50%, it indicated that there was heterogeneity between studies and a random-effects model was used. Sensitivity analyses can be executed to analyze sources of heterogeneity when necessary. The mean difference (MD) was used for the measurement data, and the relative risk (RR) was used for the count data. Both were expressed with a 95% confidence interval (CI). When *P* ≤ .05, the difference was statistically significant.

## 3. Results

### 3.1. Article search and screening

**Figure 1.**
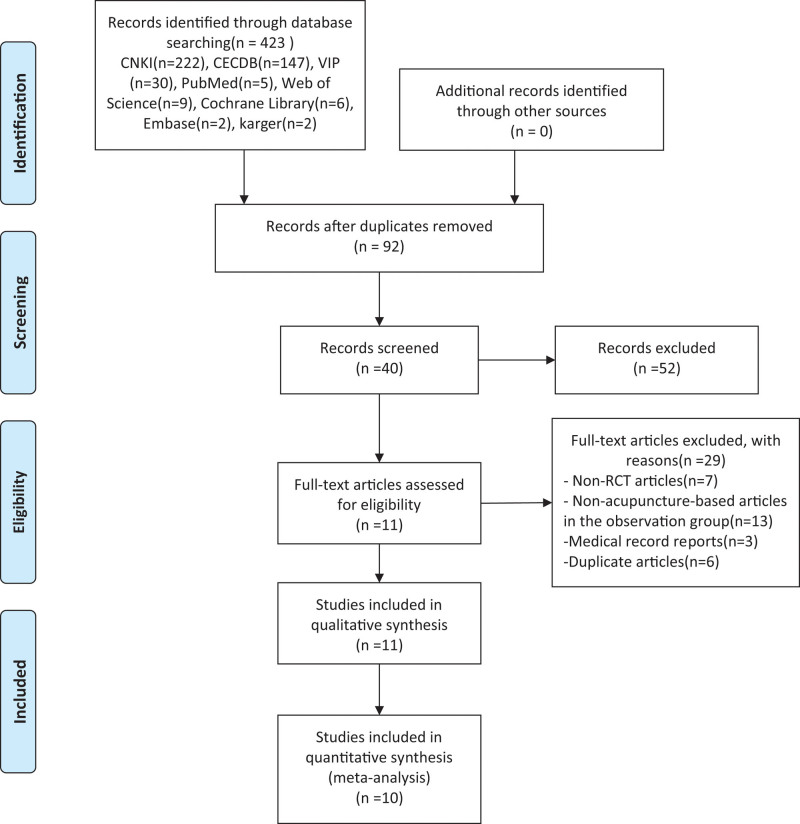
Flow chart of the trial selection process.

According to the retrieval strategy, a total of 423 articles were retrieved, including 222 articles in China National Knowledge Infrastructure Database, 147 articles in CECDB, 30 articles in VIP, 5 articles in PubMed, 9 articles in Web of Science, 6 articles in Cochrane Library, 2 articles in Embase and 2 articles in karge. Importing Note Express 3.5.0 software to remove duplicate articles, 92 articles were initially retrieved. After reading the title and abstract, 52 review articles were eliminated. After reading the full text of the remaining 40 articles, 29 articles were eliminated, including 7 non-RCT articles, 13 non-acupuncture-based articles in the observation group, 3 medical record reports, and 6 duplicate articles. Finally, 11 articles were included, all of them in Chinese (Fig. 1).

### 3.2. Essential features of included articles

Eleven incorporated studies,^[27–37]^ published between 2009 and 2022, were randomized controlled trials. A total of 781 subjects were included, including 391 in the treatment group and 390 in the control group. The articles included in the analysis are summarized in Table 1.

**Table 1:** Summary of included studies.

Study ID	Sample size (T/C)	Intervention		Frequency/course of treatment	Needle retention time (min)	Outcomes
		T	C			
Lu 2021	56/56	MA + TCM + amitriptyline	Amitriptyline	qd/4 w	15	⑤
Zhang 2021	48/48	MA + TCM + carbamazepine	Carbamazepine	qd/4 w	15	⑤
Chao 2021	33/34	MA + TCM	Carbamazepine	qd/4 w	/	③④
Song 2009	29/28	MA	Carbamazepine	qd/6 w	30	
Wang 2009	30/29	MA	Carbamazepine	qd, biw/4 w	Immediate	
Lu 2018	40/40	MA	Carbamazepine	qd/4 w	30	③④
Wang 2018	30/30	MA	Pregabalin	qd/8 w	20	⑤
Kong 2018	21/21	MA	Pregabalin	bid/2 w	30	③④
Li 2017	32/32	MA	Pregabalin	bid/8 w	Immediate	③④⑤
Wang 2014	42/42	MA + TCM	Carbamazepine	qd/4 w	30	⑤
Yang 2022	30/30	MA + TCM	Carbamazepine	qd/4 w	40	④

Outcomes: ① The total efficiency; ② VAS; ③ PPI; ④ PRI; ⑤ Adverse reactions.

C = control group, MA = manual acupuncture, qd = once a day, T = treatment group, TCM = traditional Chinese medicine, w = weekly.

#### 3.2.1. Interventions.

Among the interventions in the experimental group of the 11 articles incorporated, 6 articles used acupuncture only,^[30–35]^ 3 articles used acupuncture combined with traditional Chinese medicine,^[29,36,37]^ and 2 articles used acupuncture combined with western medicine and traditional Chinese medicine.^[27,28]^

Among the interventions in the treatment group of the incorporated 11 articles, 1 used amitriptyline,^[27]^ 7 used carbamazepine,^[28–32,36,37]^ and 3 used pregabalin.^[33–35]^

#### 3.2.2. Frequency and duration of therapy.

In terms of therapy frequency of the 11 incorporated articles, 8 articles were administered once a day,^[27–30,32,33,36,37]^ 2 articles were administered twice a day,^[34,35]^ and 1 article was punctured with plum-blossom needle once a day,^[31]^ bloodletting with 3-edged acupuncture twice a week.

Among the 11 incorporated articles in terms of course of treatment, 1 article was treated for 2 weeks,^[34]^ 7 articles were treated for 4 weeks,^[27–29,31,32,36,37]^ 1 article was treated for 6 weeks,^[30]^ and 2 articles were treated for 8 weeks.^[33,35]^

#### 3.2.3. Needle retention time.

Among the 11 included articles in terms of needle retention time, 2 articles were retained for 15 minutes,^[27,28]^ 1 was retained for 20 minutes,^[33]^ 4 were retained for 30 minutes,^[30,32,34,36]^ 1 was retained for 40 minutes,^[37]^ 2 were immediate acupuncture,^[31,35]^ and 1 did not mentioned.^[29]^

#### 3.2..4. Outcomes.

Eleven included articles all used the total efficiency rate as the observation index, and 10 articles were scored by the VAS,^[27–29,31–37]^ 5 articles were scored by the PRI and the PPI,^[29,30,34,35,37]^ and 6 articles were observed adverse reactions.^[27,28,30,33,35,36]^

### 3.3. Methodological quality assessment

**Figure 2.**
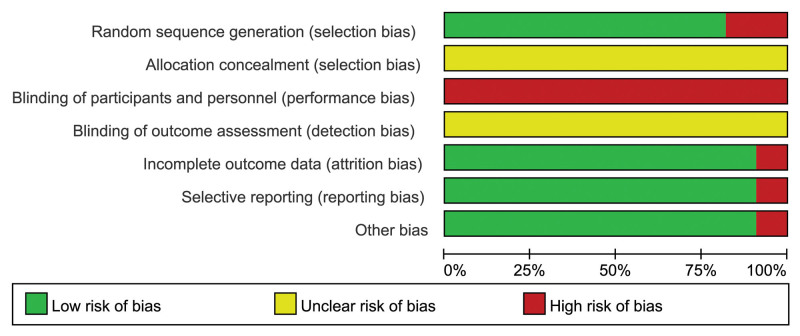
Bar chart of risk of bias.

**Figure 3.**
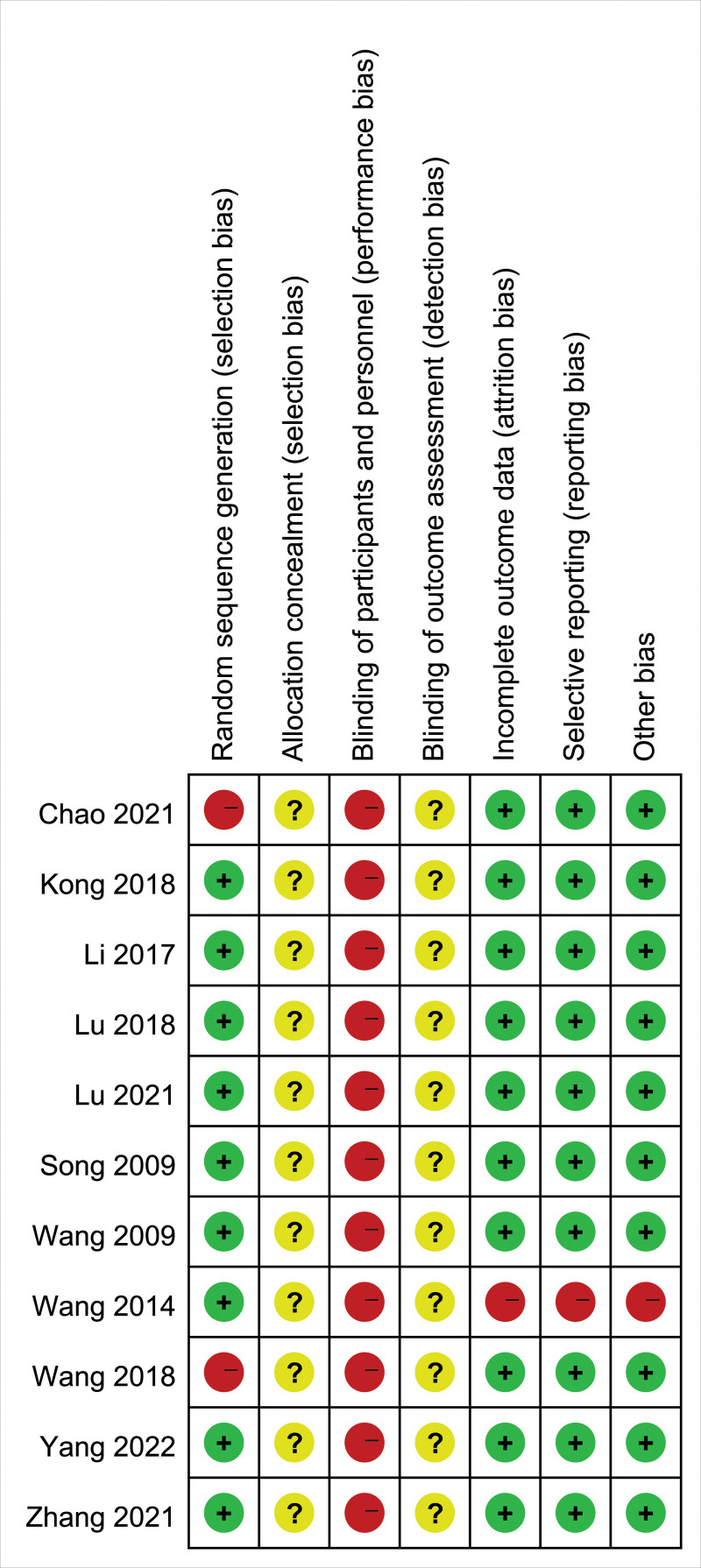
Summary chart of risk of bias.

The Risk of Bias tool was used to evaluate the methodological quality of the included articles (Figs. 2, and 3). The results showed: In terms of random sequence generation, 9 articles used the random number table method, 1 article was in the order of visits, 1 article only mentioned the random method, but did not specify the random method; Allocation concealment: none of the 11 articles mentioned allocation concealment; Blinding the subjects and researchers: none of the 11 articles were blinded; Blinding of outcome evaluators: none of the 11 articles mentioned it; Incomplete outcome data: 1 article dropped out and 12 cases were excluded before the start of the experiment, and the remaining 10 articles did not drop out, cases were excluded; Selective reporting: the 11 included articles were all complete with outcome data, and the specific results were described in detail according to their respective outcome indicators; Other sources of bias: none.

### 3.4. Meta-analysis results

#### 3.4.1. VAS.

**Figure 4.**
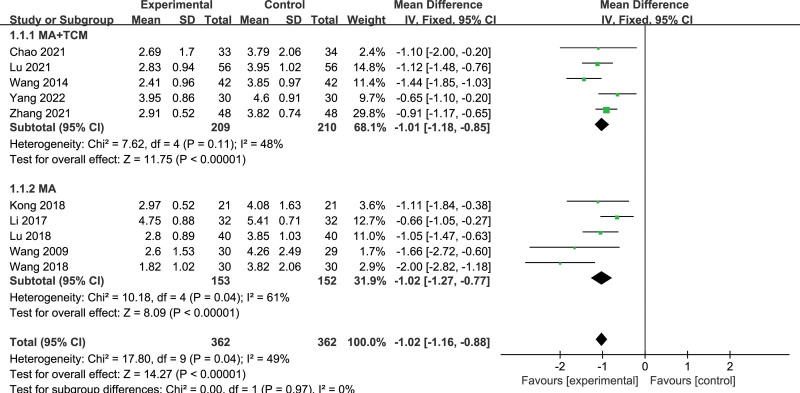
Forest plot of VAS of acupuncture versus drugs in the treatment of thalamic pain. VAS = visual analog scale.

Ten included articles reported the VAS score,^[27–29,31–37]^ and a total of 724 subjects were included, including 362 in the experiment group and 362 in the control group. The results showed that *P* = .04, *I*^2^ = 49%, and there was moderate heterogeneity (Fig. 4). Subgroup analysis was conducted in the treatment group according to different intervention methods. Heterogeneity in the manual acupuncture (MA) + traditional Chinese medicine (TCM) group did not change markedly [*P* = .11, *I*^*2*^ = 48%], while heterogeneity in the MA group increased significantly [*P* = .04, *I*^2^ = 61%]. Sensitivity analysis indicated that when Wang Shumin article^[33]^ [MD = −2.00, 95% CI (−2.82, −1.18)] was excluded, the subgroup heterogeneity decreased significantly [*P* = .24, *I*^*2*^ = 28%], and the overall heterogeneity also decreased significantly [*P* = .14, *I*^*2*^ = 34%]. The possible reason was that intervention measures were EA while the other groups were only manual acupuncture, so the heterogeneity was large. Meta-analysis results showed that acupuncture [MD = −1.06, 95% CI (−1.20, −0.91)] appeared to be more effective than drug for the treatment of thalamic pain in reducing VAS (*P* < .00001).

#### 3.4.2. PPI.

**Figure 5.**
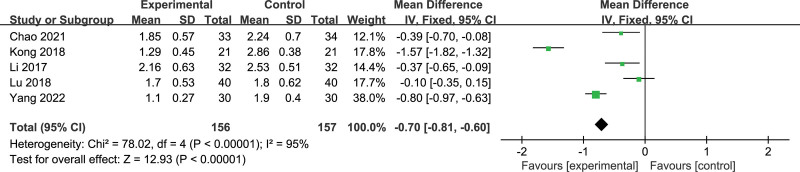
Forest plot of PPI of acupuncture versus drugs in the treatment of thalamic pain. PPI = present pain intensity score.

Five included articles reported the PPI score,^[29,32,34,35,37]^ and a total of 313 subjects were included, including 156 in the treatment group and 157 in the control group. The results showed that *P* < .00001, *I*^2^ = 95%, (Fig. 5). The heterogeneity was large. Subgroup analysis was performed according to the different intervention methods of the treatment group, and the heterogeneity of each group was still large (MA: *P* < .00001, I^2^ = 97%; MA + TCM: *P* = .02, *I*^2^ = 81%). When Kong Ying article^[34]^ [MD = −1.57, 95% CI (−1.82, −1.32)] and Yang Xiaohong article^[37]^ [MD = −0.80, 95% CI (−0.97, −0.63)] were excluded, heterogeneity decreased significantly (*P* = .24, *I*^2^ = 29%). We conclude that this may be due to greater diagnostic heterogeneity. Kong Ying has not adopted the diagnostic criteria of TCM; Yang Xiaohong did not specify the diagnostic criteria of Western medicine, and only the diagnostic criteria of Western medicine were drawn up in the inclusion criteria, so the results of the research are fairly heterogeneous. The other 3 articles indicated the diagnostic criteria of traditional Chinese and western medicine.^[29,32,35]^ Meta-analysis showed that the PPI score of acupuncture treatment for thalamic pain after stroke was lower than that of drug group [MD = −0.27, 95% CI (−0.43, −0.11), *P* = .001].

#### 3.4.3. PRI.

**Figure 6.**
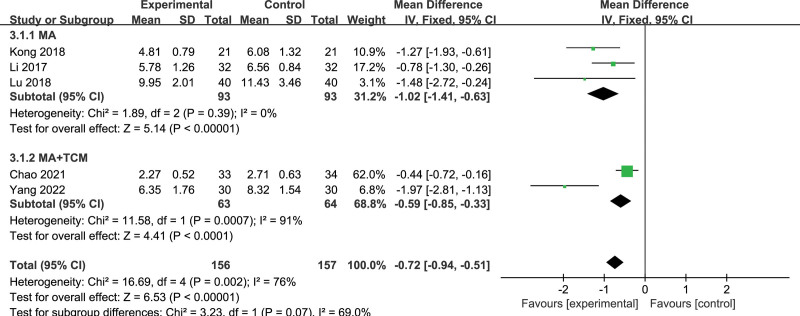
Forest plot of PRI of acupuncture versus drugs in the treatment of thalamic pain. PRI = pain rating index.

Five articles reporting PRI were included,^[29,32,34,35,37]^ and a total of 313 subjects were included, including 156 in the treatment group and 157 in the control group. The results showed that *P* = .002, *I*^2^ = 76%, there was large heterogeneity. Subgroup analysis was performed according to different intervention methods in the treatment group (Fig. 6), and the results showed that heterogeneity was minor in the MA group, while heterogeneity was enormous in the MA + TCM group (MA: *P* = .39, *I*^2^ = 0%; MA + TCM: *P* = .07, *I*^2^ = 91%). Meta-analysis results showed that manual acupuncture treatment of thalamic pain was more effective in reducing PRI than the drug [MD = −1.02, 95% CI (−1.41, −0.63), *P* < .00001].

#### 3.4.4. The total efficiency.

**Figure 7.**
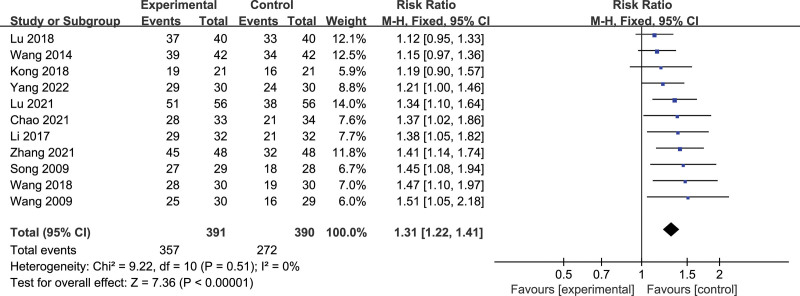
Forest plot of the total efficiency of acupuncture versus drugs in the treatment of thalamic pain.

Eleven articles reported the total efficiency,^[27–37]^ and a total of 781 subjects were involved, including 391 in the treatment group and 390 in the control group. The results showed that *P* = .51, *I*^2^ = 0%, there was less heterogeneity (Fig. 7). Meta-analysis results show that acupuncture is superior to the drug group in the total effective rate of thalamic pain [RR = 1.31, 95% CI (1.22,1.41), *P* < .00001].

#### 3.4.5. Safety evaluation.

**Figure 8.**
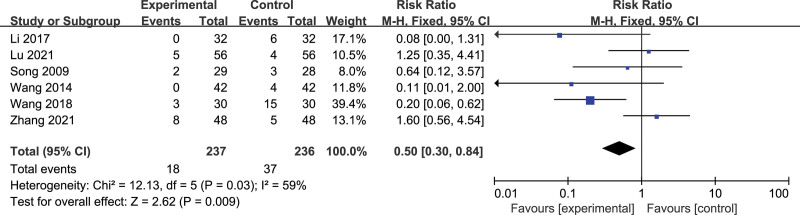
Forest plot of the total efficiency of acupuncture versus drugs in the treatment of thalamic pain.

Five articles did not mention adverse reactions.^[29,31,32,34,37]^ 6 articles mentioned adverse reactions and described them in detail,^[27,28,30,33,35,36]^ with a total of 473 cases, 237 in the treatment group and 236 in the control group. The Meta-analysis results showed (Fig. 8) that 5 articles 95% CI horizontal line intersects the invalid line,^[27,28,30,35,36]^ indicating that there was no statistical difference in adverse reactions between the treatment group and the control group, 1 article suggested that the adverse reactions in the control group were higher than those in the treatment group.^[33]^ Meta-analysis results show that there is no significant difference in safety between acupuncture and drug therapy [RR = 0.50, 95% CI (0.30,0.84), *P* = .009].

### 3.5. Publication bias

**Figure 9.**
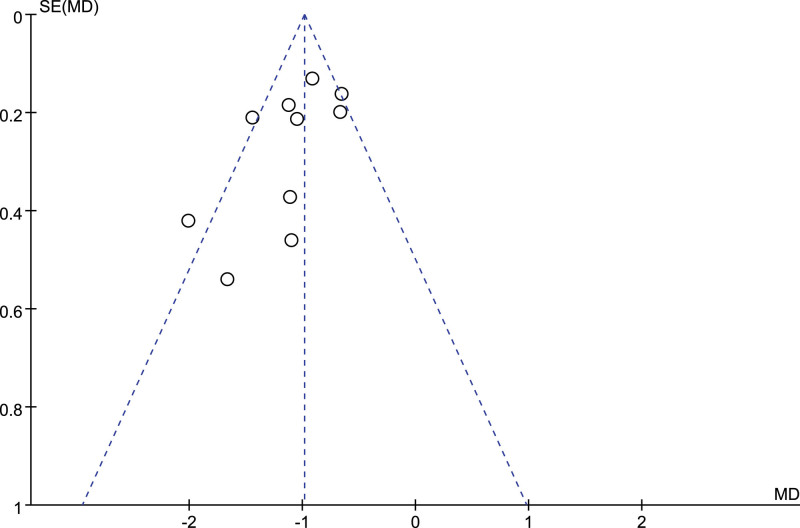
Funnel plot of VAS of acupuncture versus drugs in the treatment of thalamic pain. VAS = visual analog scale.

**Figure 10.**
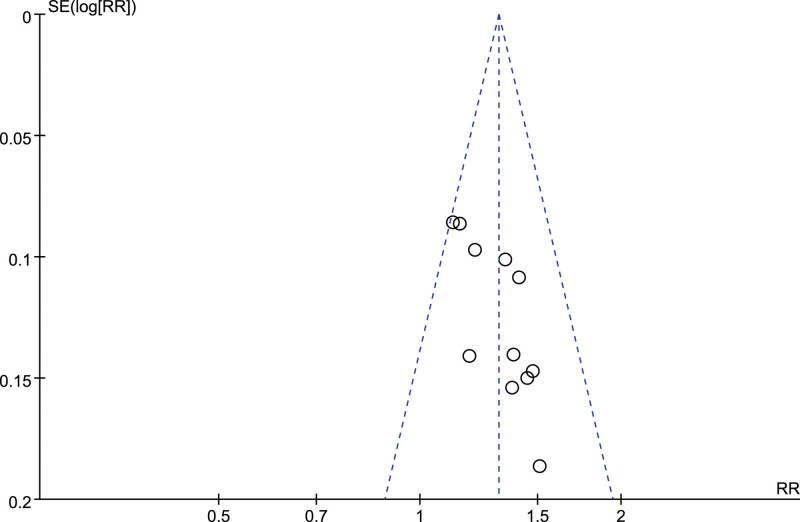
Funnel plot of the total efficiency of acupuncture versus drugs in the treatment of thalamic pain.

Publication bias was analyzed for VAS of 10 included articles and total effective rate of 11 included articles, and funnel diagrams were shown in Figures 9 and 10. Egger text based on funnel plot showed no significant publication bias (VAS: *P* = .319; the total efficiency: *P* = .197).

## 4. Discussion

### 4.1. Principal findings

A total of 781 subjects were included in this research, which included 11 RCTs. In terms of reducing patients’ pain score, there was great heterogeneity in the manual acupuncture group, which may be due to the fact that other studies in the subgroup all used manual acupuncture, while Wang research^[33]^ added EA, so there was great heterogeneity; in terms of lowering PPI score, the heterogeneity of Kong^[34]^ and Yang^[37]^ papers may be large due to the difference in diagnostic criteria, other studies all adopted the diagnostic criteria of integrated traditional Chinese and western medicine, while these 2 studies did not indicate the diagnostic criteria of TCM; in terms of reducing PRI score, manual acupuncture group had a better effect, while acupuncture combined with traditional Chinese medicine did not have a significant advantage over western medicine. The clinical experiment results of 11 articles included were all positive, and acupuncture treatment of thalamic pain was obviously superior to western medicine in terms of effective rate. In this study, it can only be concluded that acupuncture is superior to western medicine in the treatment of thalamic pain after stroke, and the intervention methods of acupuncture and acupuncture combined with TCM are better. There may be differences in results according to different observed indicators, so there is no conclusion yet.

In terms of safety, drug adverse reactions as dizziness, dry mouth, drowsiness, gastrointestinal symptoms, and adverse reactions of acupuncture for subcutaneous bleeding, strong sense of needle legacy, at the end of the withdrawal or acupuncture treatment can obviously relieve or disappear, never reported left side effects. Therefore, acupuncture therapy is safe, but there were no significant differences compared with western medicine.

We discovered that when prescribing acupuncture parameters, most doctors tend to treat 4 courses of acupuncture once a day, 6 days for a course of treatment, but the duration of needle retention is a large time span from no needle retention to 40 minutes. There are currently few fundamental or clinical studies on the duration of needle retention. Domestic studies^[39–41]^ on the duration of needle retention in patients with poststroke diseases advocate long-term needle retention. For example, Han’s team concluded that^[42]^ the longer the duration of needle retention in patients with poststroke diseases, the better. There are few external studies on the duration of needle retention.^[43]^ This reduces the repeatability of basic experiments and loses its guiding significance for clinical research and practice. Therefore, the needling time is becoming more and more important, and its regularity is worth exploring deeply.

### 4.2. Strengths and limitations

This review and meta-analysis have several advantages. Firstly, in the published systematic review articles comparing acupuncture and western medicine in the treatment of thalamic pain, there are no outcomes such as PPI, PRI, and the impact factor of the article is low. Secondly, this review included articles up to June 25, 2022, not only evaluating the efficacy and safety of acupuncture in terms of various outcome indicators, but also adding acupuncture-related parameters, such as treatment frequency, duration of treatment, needle retention time, etc, to analyze the effect of different doses on clinical outcomes related to thalamic pain. Thirdly, most of the included articles used clinical outcome measures to measure the severity and clinical efficacy of thalamic pain, and when multiple outcome measures were present, the clinician’s rating scale was given priority.

Regarding the limitations of this meta-analysis, first of all, the sample size of the involved researches was comparatively little, and there were 5 researches with no more than 60 cases, and the sample size was not estimated in all the studies. The small sample size reduces the generality of the conclusions. Secondly, the included studies were all Chinese literature. The positive rate of the results was high, and the quality of the articles was medium or low. In particular, there were uncertain risks in blind methods and allocation hiding. Thirdly, the inclusion criteria included not only manual acupuncture, but also a combination of other acupuncture methods, and the acupuncture parameters could not be unified, resulting in heterogeneity of results. Fourthly, the effectiveness was only related to the results at the end of treatment. There were only 2 follow-up studies, and the effectiveness was lower than that at the end of treatment, so there was not enough evidence to demonstrate that the efficacy of acupuncture was sustainable.

### 4.3. Implications for research and practice

In terms of the significance of the study, first of all, we believe that it is most important to expand the randomized controlled trials of large samples to convince the effectiveness and safety of acupuncture in the treatment of thalamic pain after stroke. Secondly, researchers should reduce the risk of bias as much as possible when designing experiments. For example, the random-number table method should be used in grouping rather than the order of treatment, subjects and outcome evaluators were blinded, the adverse reactions of patients were observed, long-term follow-up was conducted to observe the after effect of acupuncture. Furthermore, for the dose of acupuncture, researchers can provide longer treatment sessions and appropriately extend the duration of acupuncture retention, which may increase the effectiveness of acupuncture. Eventually, if a randomized controlled trial between acupuncture and sham acupuncture can be designed, the effectiveness of acupuncture can be better demonstrated, but due to ethical issues, it may be harder to achieve.

In terms of the significance of clinical practice, this paper can provide some theoretical support for clinical therapists.^[44]^ The pathophysiological mechanism of poststroke pain in western medicine is still unclear, and there are many reasons for the injury, including brain injury, vascular injury and destruction of inflammatory factors. Medications improve pain in 70% of stroke survivors with CPSP, with tricyclic antidepressants and antiepileptic drugs used as first-line drugs.^[45,46]^ But long-term use of antidepressants can alter the positive effects, lose their effectiveness, increase inflammation,^[47]^ long-term use of antiepileptic drugs can induce many adverse reactions and drug interactions, most frequently psychological and behavioral adverse reactions.^[48]^ Nonetheless, acupuncture is efficient for the treatment of chronic pain and is consequently a reasonable referral option.^[49,50]^ A study on the persistence of acupuncture effect in patients with chronic pain indicated that^[51]^ the effect of acupuncture did not substantially decrease after 12 months of acupuncture. Consequently, based on the comprehensive consideration of efficiency and time cost, acupuncture can be preferred in the treatment of thalamic pain.

## 5. Conclusion

In conclusion, compared with drug treatment, acupuncture can reduce the VAS, PPI and PRI scores of patients with poststroke thalamic pain, and improve the effective rate, and no serious adverse reactions have been reported so far. However, due to its blinding and risk of bias, these results need to be interpreted with caution, especially the comparison of acupuncture alone with acupuncture combined with traditional Chinese medicine, there is heterogeneity in some observation indicators. Differences in acupuncture dosage, and inconsistencies in long-term efficacy and safety. It is determined that large-scale randomized controlled trials are required to verify and track in the future.

## Author contributions

**Conceptualization:** Wenfeng Li.

**Data curation:** Wenfeng Li.

**Funding acquisition:** Shaozong Chen.

**Formal analysis:** Wenfeng Li.

**Investigation:** Wenfeng Li.

**Methodology:** Wenfeng Li.

**Project administration:** Wenfeng Li.

**Resources:** Wenfeng Li.

**Software:** Wenfeng Li.

**Supervision:** Wenfeng Li.

**Validation:** Wenfeng Li.

**Visualization:** Wenfeng Li.

**Writing – original draft:** Wenfeng Li.

**Writing – review & editing:** Shaozong Chen.
